# HomolWat: a web server tool to incorporate ‘homologous’ water molecules into GPCR structures

**DOI:** 10.1093/nar/gkaa440

**Published:** 2020-06-02

**Authors:** Eduardo Mayol, Adrián García-Recio, Johanna K S Tiemann, Peter W Hildebrand, Ramon Guixà-González, Mireia Olivella, Arnau Cordomí

**Affiliations:** Unitat de Bioestadistica, Facultat de Medicina, Universitat Autònoma de Barcelona, Bellaterra 08193, Spain; Unitat de Bioestadistica, Facultat de Medicina, Universitat Autònoma de Barcelona, Bellaterra 08193, Spain; Institute of Medical Physics and Biophysics, Medical University Leipzig, Leipzig, Sachsen 04107, Germany; Institute of Medical Physics and Biophysics, Charité Universitätsmedizin Berlin, Berlin 101179, Germany; Institute of Medical Physics and Biophysics, Medical University Leipzig, Leipzig, Sachsen 04107, Germany; Institute of Medical Physics and Biophysics, Charité Universitätsmedizin Berlin, Berlin 101179, Germany; Berlin Insitute of Health (BIH), 10178 Berlin, Germany; Laboratory of Biomolecular Research, Paul Scherrer Institute (PSI), 5232 Villigen PSI, Switzerland; Condensed Matter Theory Group, Paul Scherrer Institute (PSI), 5232 Villigen PSI, Switzerland; Bioinformatics and Medical Statistics Group, University of Vic-Central University of Catalonia, Barcelona 08500, Spain; Unitat de Bioestadistica, Facultat de Medicina, Universitat Autònoma de Barcelona, Bellaterra 08193, Spain

## Abstract

Internal water molecules play an essential role in the structure and function of membrane proteins including G protein-coupled receptors (GPCRs). However, technical limitations severely influence the number and certainty of observed water molecules in 3D structures. This may compromise the accuracy of further structural studies such as docking calculations or molecular dynamics simulations. Here we present HomolWat, a web application for incorporating water molecules into GPCR structures by using template-based modelling of homologous water molecules obtained from high-resolution structures. While there are various tools available to predict the positions of internal waters using energy-based methods, the approach of borrowing lacking water molecules from homologous GPCR structures makes HomolWat unique. The tool can incorporate water molecules into a protein structure in about a minute with around 85% of water recovery. The web server is freely available at http://lmc.uab.es/homolwat.

## INTRODUCTION

Water molecules confined inside cavities in a protein, named ordered or internal water molecules, play an essential role in the structure and function of proteins, ligand binding mechanisms, and catalytic reactions. In several proteins like G protein-coupled receptors (GPCRs), water molecules also mediate the core mechanism of activation (1–5). GPCRs are the largest family of membrane proteins with over 800 members in humans. Despite sharing a common seven α-helical transmembrane architecture and similar conformation changes upon activation (1,6), they recognize a wide diversity of extracellular signals like hormones, neurotransmitters or entire proteins. As a result, they play a key role in signal transduction and have become targets of 35% of the currently approved drugs (7). Recent studies have demonstrated that the activation of these receptors involves a specific order of internal water molecules (8). Moreover, molecular dynamics simulations have shown that internal water molecules are highly conserved among GPCRs and participate in their common activation mechanism (9). Overall, this emphasizes the important role that water molecules play in GPCR function.

Sequence homology and phylogenetic analyses have classified GPCRs into six families (or classes), namely A to F (10). Recent advances in protein engineering and structural biology have led to a rapid growth in the number of GPCR structures deposited in the Protein Data Bank (PDB). Thus, 10 years ago only 4 GPCR structures of class A had been deposited in the PDB whereas today this archive hosts 346 structures of GPCRs, 64 of which belong to unique receptor subtypes of four different GPCR classes (i.e. A, B1, C and F) (GPCRdb, http://gpcrdb.org/structure/statistics, 2020). However, technical limitations including resolution severely influence the number and certainty of solved water molecules in GPCR structures. In this context, molecular modeling can help improve and maximize internal hydration of these proteins. Current tools and methods to predict water placement in proteins (reviewed in (11)), range from knowledge-based or molecular mechanics methods to simulations approaches (e.g. molecular dynamics or Monte Carlo). One common pitfall of most of the former methods is the lack of experimental data supporting the modeled position. Another group of tools such as PyWater (12) and ProBIS H2O (12) has focused on identifying conserved or homologous water molecules in proteins using experimental data (12,13). The plethora of high-resolution GPCR structures recently deposited in the PDB (14) have simultaneously increased the number of solved waters, thus opening a door to improving the placement of internal water molecules by structural homology.

Here, we present HomolWat, a freely accessible web application (available at http://lmc.uab.cat/homolwat) aimed at incorporating internal water molecules into GPCR structures by using a molecular modeling method that borrows lacking water molecules from homologous structures. HomolWat relies on an up-to-date curated database of all internal water molecules from high-resolution structures of GPCRs deposited in the PDB. The tool uses this information to superpose water molecules from related structures in a hierarchical fashion. Water molecules that fit into receptor cavities not yet hydrated are incorporated into the model. Our method offers a novel, fast and reliable way to place internal water molecules in GPCR structures.

## MATERIALS AND METHODS

### HomolWat reference database

We have constructed an up-to-date reference database with water molecules determined in all resolved GPCR structures in the PDB (15). To ensure that coordinates have been obtained using the latest experimental and computational methods we have downloaded the structures from PDB- REDO whenever possible (16). Any non-protein molecules other than water, or GPCR orthosteric/allosteric ligands were removed from the structure. Additionally, auxiliary proteins used to assist in structure stabilization were also removed. Water molecules with low order (*B*-factor > 45 Å^2^), (17) were discarded. Water molecules were classified as internal or external based on their circular variance, following a previously reported method (18). We discarded external water molecules (those with circular variance < 0.6, computed within a radius of 10 Å around the water oxygen) for being incompatible with the membrane outside the crystal lattice. At the moment of writing HomolWat database contains 191 receptor chains from 150 high-resolution structures and 44 unique receptors, totaling 2448 internal water molecules (see http://lmc.uab.cat/homolwat/gpcr_table). The distribution of internal waters within GPCR classes and subfamilies is shown in Supplementary Figure S1.

### Implementation of the web service

HomolWat relies on a Python (v.3.7) backend that uses the Flask web framework (v1.1.1). Data for the internal water molecules is stored in a MySQL database (v.8.0.18). The web server exploits the capabilities of the popular web-based viewer NGL (v.2.0.0, (19)) for structure visualization. Preprocessing of new GPCR structures and placement of water molecules is fully automated within a routine using Python and Bash scripts. Sequence alignment is performed using Blast+ (v2.6.0+, (20)).

## RESULTS

### HomolWat pipeline

**Figure 1.**
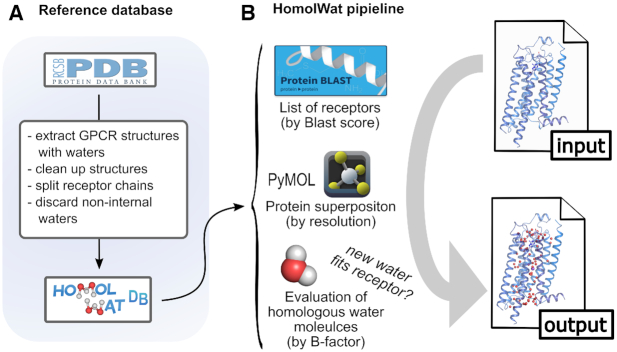
Schematic representation of the HomolWat reference database (**A**) and overall pipeline (**B**). In (B), the user provides an input 3D structure file of a GPCR—that could contain ligands or water molecules—in PDB format and obtains an output PDB file of the same structure with additional homologous internal water molecules inserted. As a first step, HomolWat extracts the protein sequence from the PDB file and runs Blast+ on all receptor sequences in HomolWatDB. This provides a list of receptors sorted by Blast score from high to low sequence homology. Subsequently, HomolWat uses this list to assess if a water molecule fits in the receptor, considering previously inserted water molecules. This assessment is performed for every water molecule across all receptors within chosen Blast+ score, and across every structure for each receptor.

HomolWat protocol is schematically represented in Figure 1. The input of HomolWat is a file with the 3D structure in the PDB format (alternatively the use can select a PDB id and a specific chain) whereas the output is the same structure containing homologous internal water molecules obtained from the HomolWat reference database. This hydrated structure can be visualized interactively using NGL viewer (19) and downloaded for further use. The overall HomolWat protocol for water molecule placement contains steps as follows: First, HomolWat uses Blast+ to perform a multiple sequence alignment of the input structure sequence against all GPCR sequences from receptors hosted in HomolWatDB, resulting in a list of homologous structures with water molecules sorted according to their Blast+ score. The user is then required to choose the GPCR functional state (i.e. inactive or active) in order to prioritize water molecules present in active or inactive structures. The user can decide whether to try to incorporate or not a sodium ion near the conserved residue D2.50 (numbering following Ballesteros and Weinstein scheme (21)) present in most inactive structures (8,22) from the structure with the highest sequence identity and best resolution. The ion is introduced when there are no water molecules within a radius of 2.1 Å or protein atoms within 1.8 Å (23). Moreover, users have the option to run popular energy-based method Dowser+ (24) alongside the main HomolWat protocol and incorporate predicted water molecules that do not overlap with homologous waters. This option becomes more useful when few homologous water molecules exist. In addition, using a Blast+ score threshold, the user can limit the incorporation of water molecules from close homologs only. Next, Homolwat performs a global structural alignment of homologous structures to the query structure in descending order of Blast+ score and from highest to lowest resolution using the *align* and *super* functions of the visualization software PyMol (v.2.0.5, (25)). Subsequently, the position of each water molecule is refined through a local structural alignment of residues around 10 Å using the *super* function and only those waters with a RMSD in the local structural alignment up to 2.0 Å are kept. Waters are assessed one by one in increasing order of B-factor and incorporated into the model should they not clash with atoms from the query model or already incorporated water molecules (distance cutoff of 2.4 Å, (26)). The structure solvated with internal waters is shown interactively using an embedded NGL viewer (19) that also shows the source structure (PDB id, chain and Uniprot entry name) of each water molecule. The solvated structure, a list with the incorporated water molecules and a PyMOL session with the structures used for water positioning can be downloaded for further usage.

### Test case

**Figure 2.**
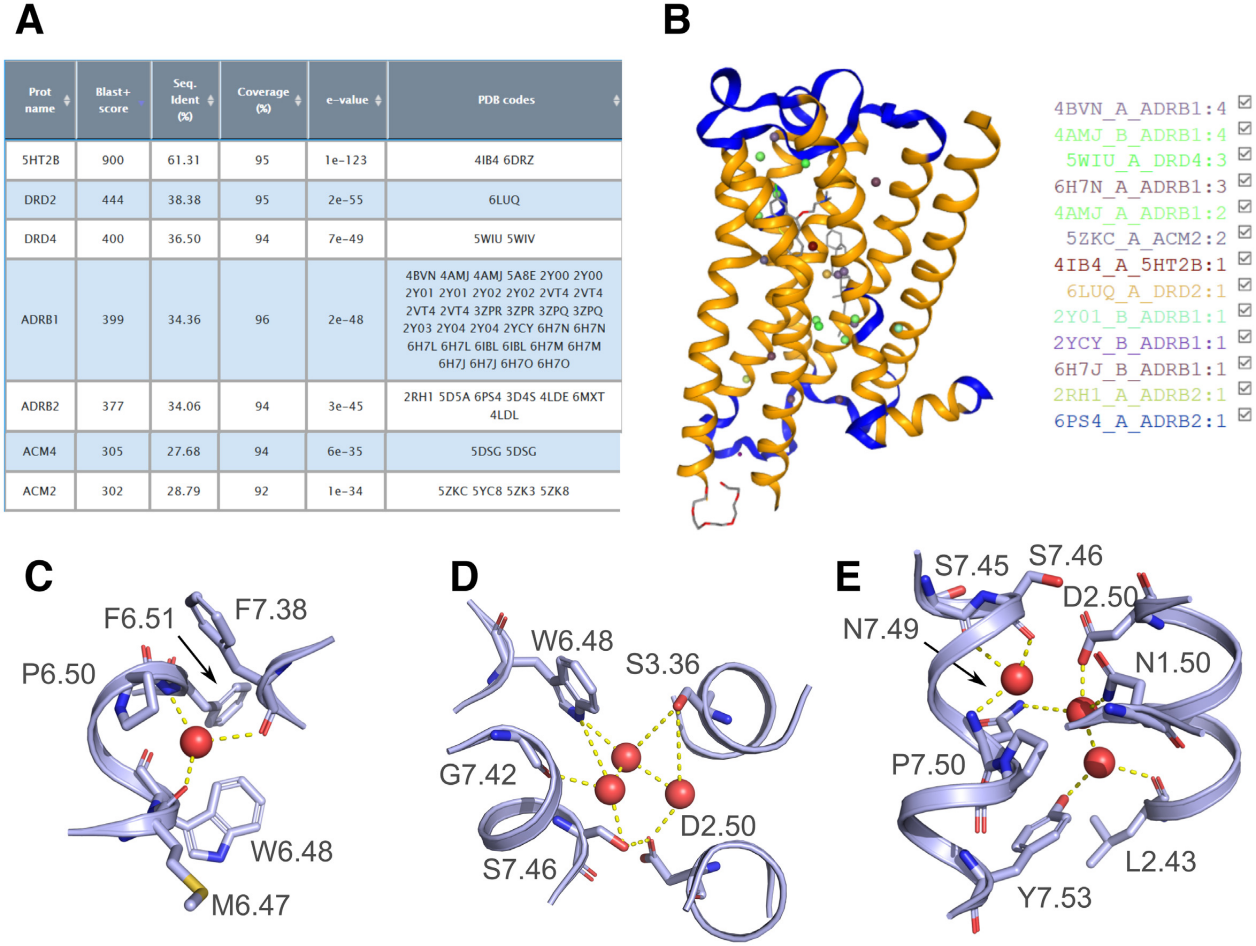
HomolWat test case: the crystal structure of the inactive serotonin 5-HT_2A_ receptor (PDB id 6A94) without internal water molecules. (**A**) Screenshot of the results for the Blast+ alignment to receptors in HomolWatDB. (**B**) Screenshot of the output PDB structure with the introduced internal water molecules displayed in NGL viewer. (**C-E**) Details of the clusters of water molecules associated to functionally important residues at different levels ranging from the orthosteric site to the G-protein binding crevice upon incorporation to its receptor core using HomolWat. The receptor is shown in light blue cartoons and water molecules are displayed as red spheres.

To illustrate the use of HomolWat, we used our tool to place internal water molecules into the recently resolved crystal structure of the inactive serotonin 5-HT_2A_ receptor at 2.9 Å resolution (PDB id 6A94, (27)) where no internal water molecules could be determined. We selected chain A of this structure using the dropdown menu that loads preprocessed structures without fusion protein, nanobodies or other molecules used for crystallization purposes. We specified i) that the structure is in an inactive state, ii) that we would like to add the conserved sodium, iii) that we do not want to use Dowser+ to predict additional waters and that we will allow structures with a Blast+ score threshold of 300 (i.e. include all amine receptors). In about 15 seconds, HomolWat incorporated 25 water (Figure 2A–B) molecules from the homologous 5-HT_2B_ receptor (61% sequence identity, 1 water), dopamine D2 receptor (38%, 1 water), dopamine D4 receptor (37%, 3 waters), β_1_-adrenergic receptor (34%, 16 waters), the β_2_-adrenergic receptor (34%, 2 waters) and the muscarinic M_2_ receptor (29%, 2 waters). The hydrated model contains the conserved water at the large proline-associated kink in transmembrane helix 6 (3) along with other known functional water molecules (4,9) at regions that span from near the orthosteric site to more cytoplasmic locations near residues N1.50, D2.50, N7.49 and Y7.53 (3,9) (Figure 2C–E). Interestingly, HomolWat could not place the sodium ion as the mutated protein used for crystallization included a mutation (i.e. S3.39K) near residue D2.50, which introduces a large sidechain that hampers Na^+^ binding.

### Validation

**Figure 3.**
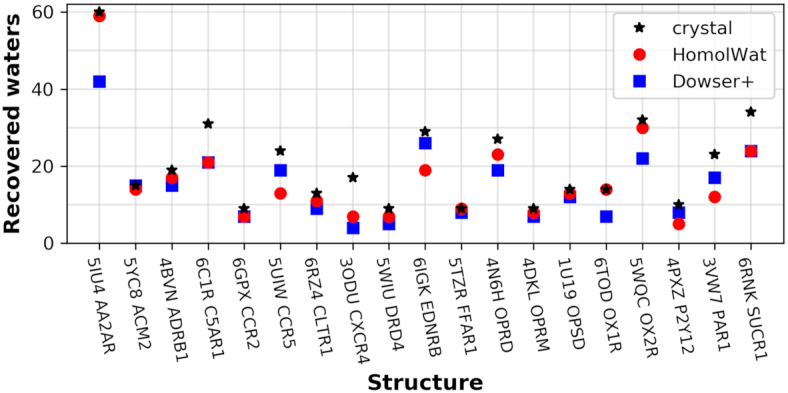
Validation of HomolWat and comparison to Dowser+. Number of water molecules recovered using Homolwat (red squares) and Dowser+ (blue dots) upon removal of the originally resolved water molecules (black star) in each PDB structure (mapped to their Uniprot accession code).

To validate the ability of HomolWat to introduce internal water molecules, we evaluated the percentage of recovery using a set of 19 Class A structures accounting for at least one unique structure from any GPCR with resolution <2.8 Å and more than five internal water molecules, where we had previously removed all water molecules. To avoid redundancy, we excluded from the reference dataset the respective water molecules of those structures included in the test set. The results were compared to Dowser+ (24) predictions, which we chose, among various alternatives (see (11)), as a representative software able to find cavities in proteins and solvate them according to an energy criterion. HomolWat places a median of 86 molecules per receptor, consistent with the number of experimentally determined water molecules within the protein core of rhodopsin (28). Figure 3 shows how many of the original internal water molecules for the tested 19 PDB structures were recovered by HomolWat using a cutoff radius <2 Å. The percentages of recovery range between 41.2 and 100%, with a median of 84.6% (see Supplementary Table S1) and the average distance between original and recovered water is 0.68 Å (see Supplementary Table S2). Larger recoveries are obtained for receptors with many homolog high-resolution structures, whereas poor recovery is obtained for receptors with a small number of determined structures with water molecules and low homology to other receptors. Our method outperforms previously described knowledge-based methods like Dowser+ (24).

## CONCLUSIONS AND FURTHER DIRECTIONS

HomolWat is a web application to introduce internal water molecules in GPCR structures using resolved water molecules from homologous structures. The tool uses a database of GPCR structures containing internal water molecules to place homologous water positions into GPCR models or experimental structures with few or no internal waters. Due to the foreseeable increase in the number of resolved high-resolution GPCR structures, HomolWat will be able to use more templates, hence increasing its current performance. Better GPCR models that explicitly introduce internal water molecules may for instance improve docking calculations or molecular dynamics simulations. HomolWat has been successfully applied as part of the pipeline used in the GPCRmd project (http://gpcrmd.org), a community-driven effort to create the first open, interactive and standardized database of GPCR molecular dynamics simulations (29). As shown in our test benchmark, HomolWat water placement pipeline showed a median recovery of 83%. The fact that HomolWat employs experimental knowledge to perform molecular modeling gives a clear competitive advantage to our tool when compared to methods based on energy calculations such as Dowser+ (24), WaterMap (30) or Waterdock (31). Due to its automated fashion, this tool could expand to target water molecule placement in other protein families.

## DATA AVAILABILITY

HomolWat is a web server freely accessible at http://lmc.uab.es/homolwat. The reference database can be obtained on request or downloaded from the website. The source code is available at https://github.com/EMayol/HomolWat.

## Supplementary Material

gkaa440_Supplemental_File

## Notes

Present address: Johanna K.S. Tiemann, Linderstrøm-Lang Centre for Protein Science, Department of Biology, University of Copenhagen, Kobenhavn, 2200, Denmark.
