# Supplementary material for: HomolWat: a web server tool to incorporate ‘homologous’ water molecules into GPCR structures
Source: Nucleic Acids Res. 2020 Jun 2;48(W1):W54–9. doi: 10.1093/nar/gkaa440 (PMC7319549; doi:10.1093/nar/gkaa440)
Supplement: gkaa440_Supplemental_File [file gkaa440_supplemental_file.pdf]

Crystal			HomolWat			Dowser+		
**Receptor**	**PDB id**	**Internal water molecules**	**Internal water molecules**	**Recovered water molecules**	**(%)**	**Internal water molecules**	**Recovered water molecules**	**(%)**
OPSD	1U19	14	72	13	92.9	88	12	85.7
CXCR4	3ODU	17	92	7	41.2	72	4	23.5
PAR1	3VW7	23	72	12	52.2	66	17	73.9
ADRB1	4BVN	19	102	17	89.5	79	15	78.9
OPRM	4DKL	9	88	8	88.9	66	7	77.8
OPRD	4N6H	27	86	23	85.2	66	19	70.4
P2Y12	4PXZ	10	65	5	50	84	8	80.0
AA2AR	5IU4	60	107	59	98.3	64	42	70.0
FFAR1	5TZR	9	71	9	100	48	8	88.9
CCR5	5UIW	24	82	13	54.2	80	19	79.2
DRD4	5WIU	9	87	7	77.8	71	5	55.6
OX2R	5WQC	32	103	30	93.8	85	22	68.8
ACM2	5YC8	15	98	14	93.3	72	15	100.
C5AR1	6C1R	31	89	21	67.7	73	21	67.7
CCR2	6GPX	9	78	7	77.8	41	7	77.8
EDNRB	6IGK	29	82	19	65.5	101	26	89.7
SUCR1	6RNK	34	62	24	70.6	56	24	70.6
CLTR1	6RZ4	13	77	11	84.6	86	9	69.2
OX1R	6TOD	14	102	14	100	73	7	50.0
**Median**			**86**		**84.6**	**72**		**73.9**

Supplementary Table 1. Recovery of water molecules using HomoWat and Dowser+ in the test set. The table lists the number of determined internal water molecules for each PDB entry, followed by the number of incorporated waters and the number and percentage of recovered waters within a radius of up to 2.0 Å relative to the resolved water.

Crystal		Homolwat		Dowser+	
**Receptor**	**PDB**	**Average distance**	**Standard deviation**	**Average distance**	**Standard deviation**
OPSD	1U19	0.43	0.26	0.64	0.27
CXCR4	3ODU	0.84	0.41	1.5	0.16
PAR1	3VW7	1.24	0.51	0.68	0.35
ADRB1	4BVN	0.46	0.34	0.89	0.44
OPRM	4DKL	1.04	0.59	1.16	0.36
OPRD	4N6H	0.93	0.55	0.78	0.46
P2Y12	4PXZ	1.33	0.42	0.57	0.4
AA2AR	5IU4	0.3	0.32	0.83	0.54
FFAR1	5TZR	0.27	0.07	0.5	0.42
CCR5	5UIW	0.83	0.56	0.78	0.47
DRD4	5WIU	0.34	0.2	0.75	0.49
OX2R	5WQC	0.58	0.43	0.73	0.52
ACM2	5YC8	0.37	0.31	0.84	0.54
C5AR1	6C1R	1.05	0.63	0.71	0.63
CCR2	6GPX	0.68	0.31	0.77	0.55
EDNRB	6IGK	1.11	0.53	0.66	0.49
SUCR1	6RNK	0.62	0.53	0.73	0.58
CLTR1	6RZ4	1.33	0.45	1.22	0.4
OX1R	6TOD	0.28	0.17	0.45	0.31
**Average**		**0.68**	**0.42**	**0.75**	**0.46**

Supplementary Table 2. Distance between the positions of incorporated and original water molecules using HomoWat and Dowser+ on the test set. The table lists the average distances for all molecules in the PDB entry and the standard deviation.

Supplementary Figure 1. Distribution of structurally resolved internal water molecules within GPCR families and subfamilies. The cumulative number of water molecules per receptor (shown in parenthesis) is represented by circle size. Circular tree adapted from a radial cluster dendrogram of a D³ Data-Driven notebook (1).

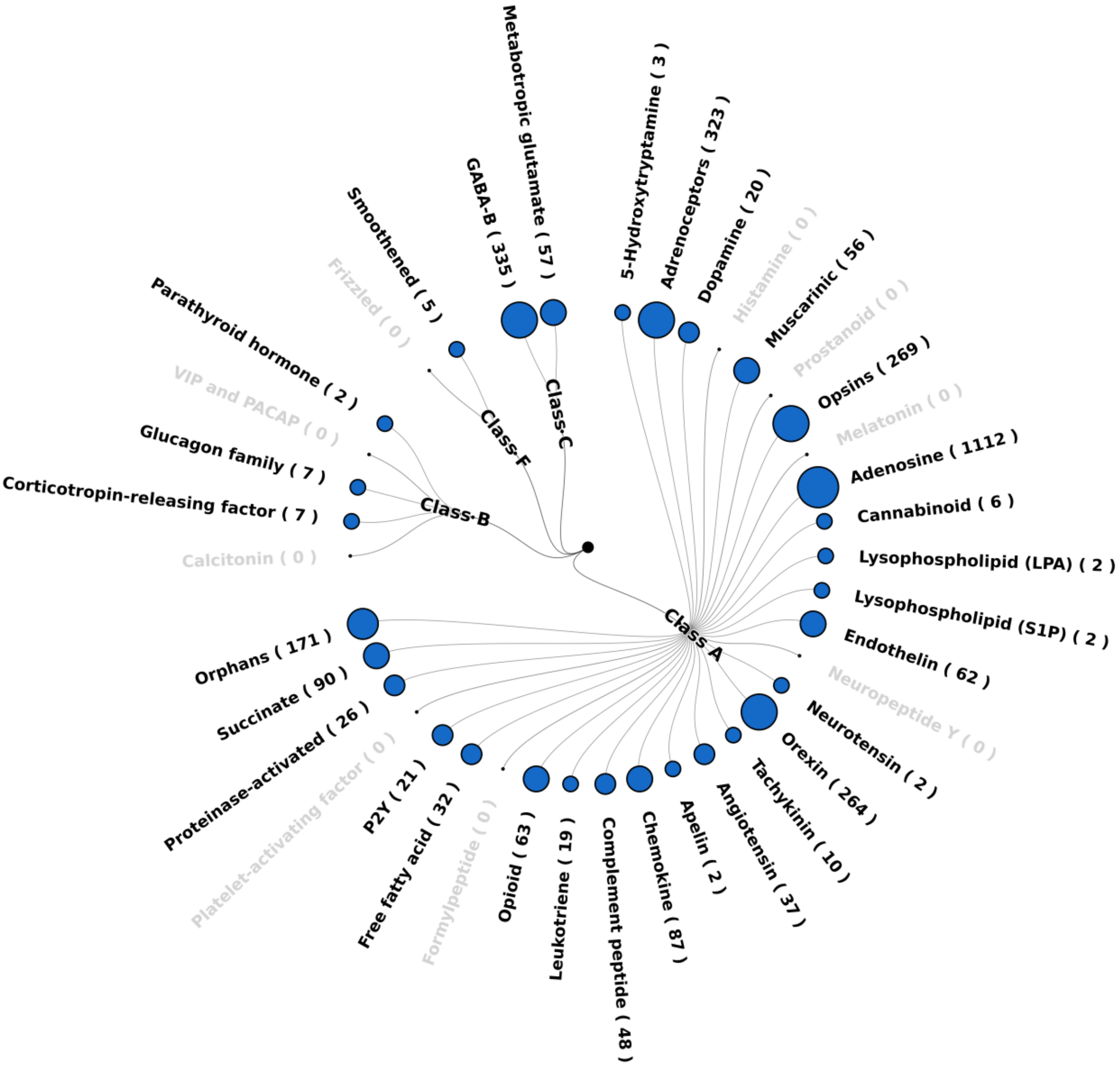

REFERENCES

1. Bostock.M.. Ogievetsky.V. and Heer.J. (2011) D3: Data-Driven Documents. IEEE Trans. Vis. Comput. Graph.. 17. 2301–2309.
